# Systematic Review and Meta-Analysis of Correlation of Progression-Free Survival-2 and Overall Survival in Solid Tumors

**DOI:** 10.3389/fonc.2020.01349

**Published:** 2020-08-14

**Authors:** Simon Chowdhury, Paul Mainwaring, Liangcai Zhang, Suneel Mundle, Eneida Pollozi, Alexander Gray, Mark Wildgust

**Affiliations:** ^1^Guy's, King's, and St. Thomas' Hospital, London, United Kingdom; ^2^Xing Technologies, Sinnamon Park, QLD, Australia; ^3^Janssen R&D, Raritan, NJ, United States; ^4^Janssen Global Services LLC, Raritan, NJ, United States; ^5^Idea Pharma, New York, NY, United States; ^6^Idea Pharma, Cranfield, United Kingdom

**Keywords:** PFS2, OS, second disease progression, surrogate clinical endpoint, time to progression on second therapy

## Abstract

**Background:** Using progression-free survival (PFS)2, time from randomization to 2nd disease progression or death, is proposed as a surrogate for overall survival (OS) in oncology clinical trials. We used published data from solid tumor trials to assess whether PFS2 and OS are correlated.

**Methods:** A literature search identified studies that reported PFS, PFS2, and OS. Two reviewers screened for eligibility, and documented PFS2, PFS or time from 1st to 2nd disease progression or death and OS. Correlation between PFS2 and OS was assessed using: (1) Kendall's Tau + Pearson's correlation coefficient in randomized controlled trials (RCTs); (2) Meta-analysis with the random effects model to compute the pooled correlation of PFS2 and OS.

**Results:** Overall, 133 studies met search criteria, 15 (28 arms) had complete PFS2 and OS data in ovarian, gastric, colorectal, prostate, lung, renal and breast cancers. A positive correlation for PFS2 and OS was found for all 15 studies (Kendall's Tau = 0.7 [95% CIs 0.54, 0.78]); 10 RCTs (Pearson's correlation coefficient = 0.86); and meta-analysis from 7 trials (pooled Spearman's correlation coefficient = 0.84 [*p* = 0.0001; 95% CIs 0.71, 0.96]).

**Conclusions:** In this retrospective analysis PFS2 strongly correlates with OS supporting the use of PFS2 to measure long-term clinical benefit when OS cannot be assessed.

## Introduction

Overall survival (OS) is recognized as the definitive endpoint in oncology clinical trials. However, time for OS data to mature can be protracted, costly, and delay effective treatments (1). Progression-free survival (PFS) is seen before OS (2, 3), but often PFS does not correlate with OS because of subsequent lines of therapy during the course of cancer treatment (2, 4). The impact of subsequent treatments can mask the effects of first-line treatment such that OS benefits are not always observed in clinical studies despite significant improvements in PFS. Also, occasionally, first line therapies can have short PFS but extend OS by sensitizing tumor cells to second-line agents (5). In contrast, occasionally longer duration of PFS on study treatment is found to correspond to longer OS. In patients with platinum-sensitive, recurrent ovarian cancer, compared with placebo treatment, niraparib significantly extended the median PFS, and this longer PFS was associated with longer median OS (6).

To address these issues, the European Medicines Agency (EMA) encourages the use of the time from randomization to initial experimental therapy to tumor progression on the next line of treatment or death from any cause (PFS2) to evaluate benefit over two lines of treatment (7).

The retrospective analysis included in this report evaluated the strength of correlation between PFS2 and OS in solid tumor oncology trials across multiple tumor types.

## Methods

### Search Strategy

An electronic literature search of Global Data was conducted for all oncology trials published from January 1, 2000 to August 10, 2018 that included the term PFS2. Global Data consisted of original journal publications, online articles, scientific meeting presentations, 90 clinical trials registries including ClinicalTrials.gov and EuroDACT, and other published reports.

### Study Selection

Individual citations were downloaded, and corresponding abstracts and full-text articles were retrieved from PubMed and Google Scholar. Publications were examined by two authors (LC, SM) for the following inclusion criteria: patients had confirmed solid tumors and were receiving treatment; outcomes measured included PFS2 or PFS and time to second progression (TTP2).

### Data Extraction

Summary data (registration identifier, and trial acronym), interventions and target population, indication, metastasis; endpoints; definitions of PFS, PFS2, TTP were extracted from cancer clinical trials reporting PFS2 (or PFS + TTP2) and OS. Out of 25 studies, four did not provide consistent definitions of PFS2 or TTP2; median survival times of PFS2 were calculated based on the availability of the medians of PFS and TTP2.

### Sensitivity and Publication Bias

The relative influence of each study was evaluated by omitting studies one at a time. Meanwhile, funnel plots were used to visualize the potential publication bias. Begg's and Egger's tests were used to evaluate the asymmetry of the funnel plot (8, 9).

### Statistical Analysis

#### Impute Medians of PFS2

PFS + TTP2 or PFS2 values were combined for different cancer types and treatment arms in our analysis. To compute the median PFS2 from PFS and TTP2, the exponential assumptions were imposed for PFS and TTP2.

#### Correlation Between PFS2 and OS

To investigate the correlation between PFS2 and OS, numerical and graphical methods were used to summarize and examine the original and imputed data using two methods. The first was based on data reported for 28 study arms in 15 studies. The second was based on randomized multi-arm studies only.

##### Method 1

Unweighted pooled estimates of PFS2 and OS were computed using median values reported or predicted for each treatment group in the included studies. The Kendall rank coefficient is non-parametric, and often used as a test statistic to establish whether two variables may be regarded as statistically dependent. Here, it is used to measure the correspondence between the ranking of PFS2 and OS. The total number of possible pairings of PFS2 and OS is $\frac{n\left(n-1\right)}{2},$ where *n* is the size of PFS2 and OS. The Kendall's Tau correlation coefficient is calculated as follows

$$\begin{array}{l} \tau =\frac{1}{n\left(n-1\right)}\underset{i\neq j}{\sum }sgn\left(PFS2_{i}-PFS2_{j}\right)\left(OS_{i}-OS_{j}\right). \end{array}$$

Kendall's Tau point estimate and 95% CI were used to summarize the correlation based on the pooled randomized controlled trials from all clinical indications together; the Kendall's Tau correlation τ is translated into Pearson's correlation ρ to evaluate the strength of the relationship (10):

$$\begin{array}{l} \rho =\sin\left(\frac{\pi }{2}*\tau \right). \end{array}$$

Pearson's correlations ranging from 0.3 to 0.5, 0.5 to 0.7 or >0.7 were classified as weak, moderate and strong, respectively.

##### Method 2

Within each indication represented by a total ≥2 arms across multiple randomized studies, Spearman's rank correlation coefficient was used to assess the dependence between medians of PFS2 and OS (11, 12). Within each indication, Spearman's rank correlation coefficient was used to assess the dependence between PFS2 and OS; subsequent meta-analysis with fixed effect or random effects models (13, 14) was used to compute the pooled correlation of PFS2 and OS in solid tumors. To be more specific, the fixed-effects model assumes that all studies along with their effect sizes stem from a single homogeneous population with underlying correlation of PFS2 and OS being the same; while, random-effects model assumes the effects are different due to tumor types, but from the same distribution. Performing a meta-analysis of correlations is to combine correlations from different studies into one pooled correlation estimate. When pooling correlations, the Fisher's r-to-z transformation for correlation was used to obtain accurate weights Conducting Meta-Analyses in R with the Metafor Package for each study based on the study sample size.

## Results

### Literature Review

**Figure 1:**
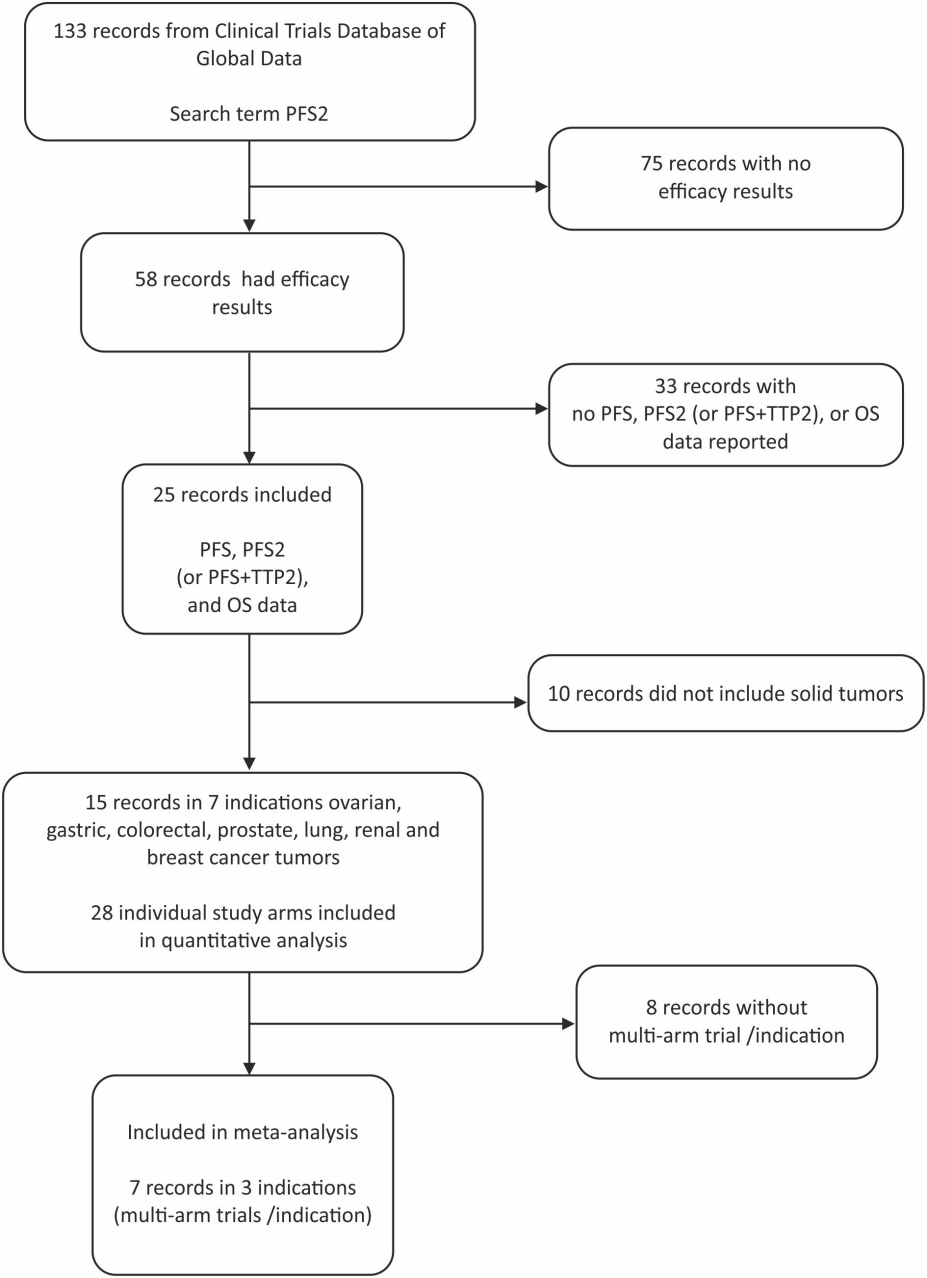
Prisma chart.

Of the 133 studies identified by the search term PFS2 (Figure 1), 75 did not report efficacy outcomes. Of the 58 that did, 25 were eligible to be screened for PFS, PFS2 (or PFS + TTP2), and OS data. Complete PFS2 and OS data sets were reported in 15 trials, for a total of 3368 patients distributed among 28 individual arms (treatment or control). Seven cancer types (ovarian, gastric, colorectal (CRC), prostate, non-small cell lung (NSCLC), renal and breast) were represented (Table 1). All cancer types except mCRPC were represented by 2 arms. Three cancers (breast, CRC, and NSCLC) that were represented in ≥1 randomized study with ≥2 arms across were included in the meta-analysis.

**Table 1:** Solid tumor studies included in PFS2 to OS correlation analysis.

**TUMOR TYPE Trial identifier**	**Study arm**	***n***	**Median PFS2 months**	**Median OS months**
**BREAST**				
OlympiAD (15, 16)	1	102	9.9	16.7
	2	103	15.3	21.8
	3	48	8.3	15.2
	4	49	9.6	21.1
HAT (17)	1	39	19.8	36.8
	2	45	19.6	54.0
**COLORECTAL**				
CRAFT (15)	1	52	13.5^*^	27.4
CAIRO (18)	1	279	8.5	18.1
	2	278	11.7	21.6
REVERCE (19)	1	50	1.8	11.6
	2	51	5.2	17.4
ROCKET (20)	1	18	4.5	5.0
**GASTRIC**				
GDCT0014765 (21)	1	27	10.2^*^	16.0
	2	32	11.8^*^	22.0
**mCRPC**				
GDCT0290285 (22)	1	59	14.3	22.1
**NSCLC**				
ULTIMATE (23)	1	57	8.4^*^	9.9
	2	13	6.5^*^	11.4
	3	21	10.6^*^	11.4
AvaALL (24)	1	245	18.9^*^	27.0^*^
	2	240	16.5^*^	24.8^*^
ASPIRATION (25)	1	93	14.1	31.0
	1	176	14.9	31.0
KEYNOTE-024 (26, 27)	1	151	8.6	14.5
BUCiL (28)	1	120	15.0^*^	17.7
**OVARIAN**				
TRINOVA-1 (29)	1	458	10.9	18.3
	2	461	12.5	19.3
**RENAL CELL CARCINOMA**				
ROPETAR (30)	1	49	14.5	18.5
	2	52	20.2	35.0

mCRPC, metastatic castration-resistant prostate cancer; NSCLC, non-small cell lung cancer.

* *Calculated*.

### Correlation of PFS2 and OS

#### All Studies

**Figure 2:**
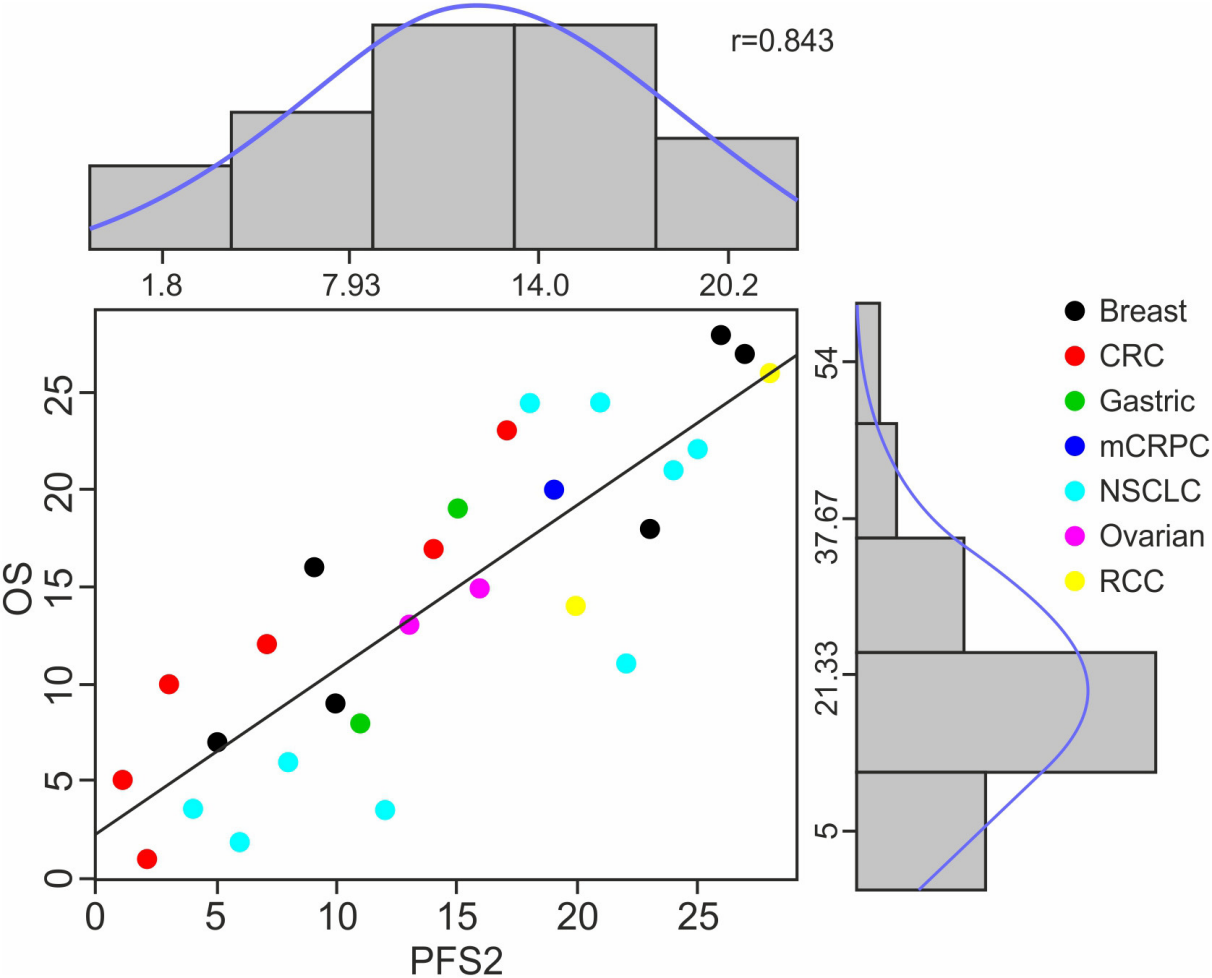
Distribution of Raw Data, Ranks and Spearman's Rank Correlation of PFS2 to OS. The scatter plot shows the Spearman's ranks and correlation details between median PFS2 and OS for 28 treatment arms among 7 cancer types. Each color represents a specific cancer type; each dot corresponds to a treatment arm. The X axis numbers represent the median PFS2 in each study in rank order and Y axis median OS for each study also in rank order. The histograms and density curves (blue) show the frequency and distribution of the raw median survival times in PFS2 and OS, respectively.

The ranges of median PFS2 and OS based on pooled data from all studies are (1.8, 20.2) and (5, 54), respectively, and the Spearman's rank correlation of 0.843 is noted. (Figure 2) A strong positive correlation between PFS2 and OS was confirmed, with a correlation of 0.70 using Kendall's Tau where a value of 0 is no relationship and 1 is a perfect correlation, Kendall's tau = 0.70 corresponds to a Pearson's correlation of 0.86 showing a strong correlation (>0.7) of OS and PFS2 as continuous variables.

#### Single Tumor Types

The correlation between PFS2 and OS was assessed for 6 individual tumor types represented by at least 2 treatment arms. A strong Spearman's rank correlation was observed within each tumor type (Table 2).

**Table 2:** Spearman's rank correlation within tumor types.

	**Number of studies (# of arms)**	**Correlation**	**95% CI**
Breast	2 (6)	0.8857	0.8642; 0.9072
Colorectal	4 (6)	0.9429	0.9348; 0.9509
Gastric	1 (2)	1.0000	
NSCLC	5 (9)	0.6891	0.6583; 0.7199
Ovarian	1 (2)	1.0000	
RCC	1 (2)	1.0000	

*NSCLC, non-small cell lung cancer; RCC, renal cell carcinoma*.

#### Meta-Analysis

**Figure 3:**
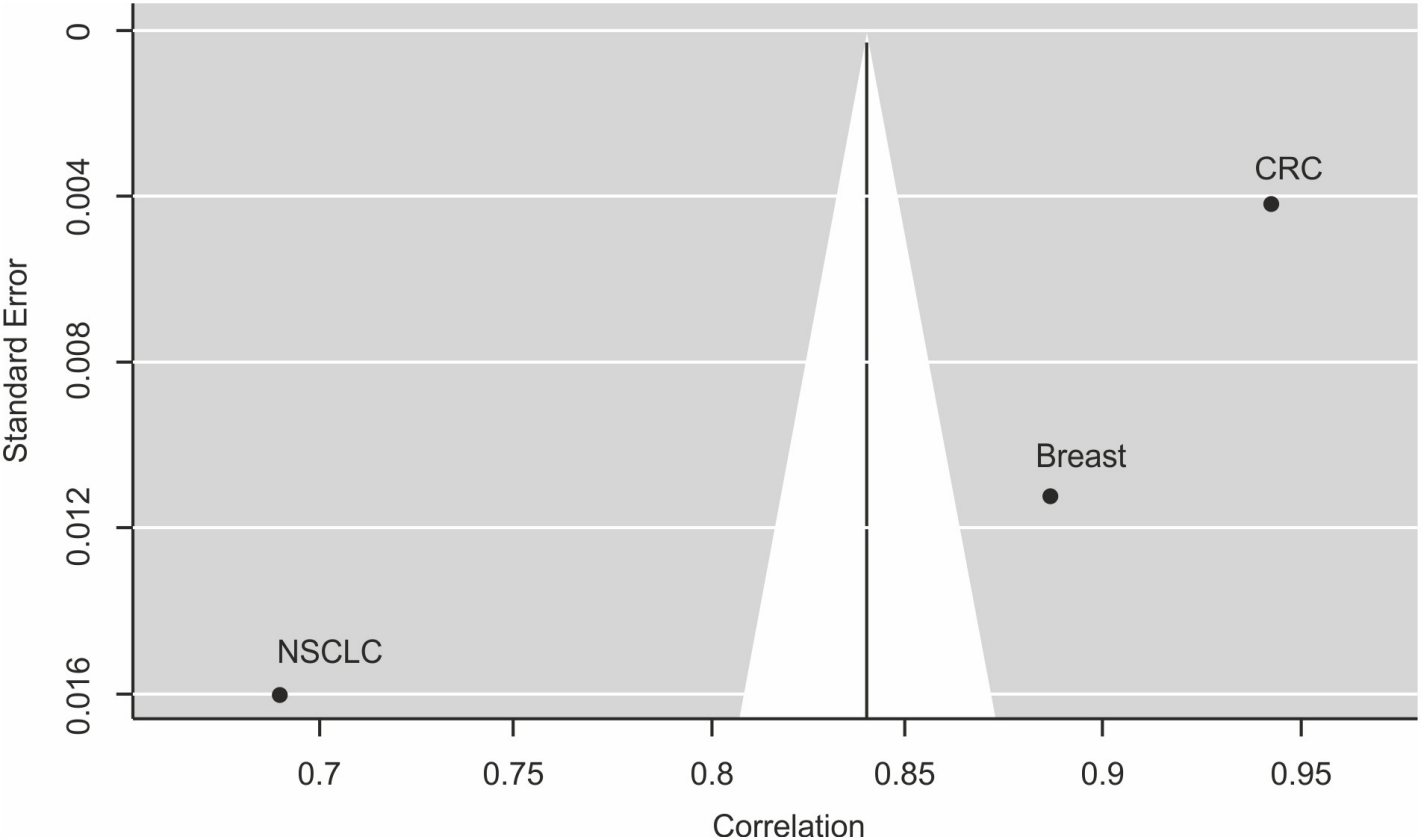
Funnel plot for publication bias analysis of the correlation between PFS2 and OS using the random effect model. This funnel plot is based on the three cancer types investigating the correlation between PFS2 and OS in meta-analysis. The X-axis stands for the correlation and the Y-axis is the standard error for each of the studies. Begg's and Egger's tests were used to evaluate the asymmetry of the funnel plot.

A meta-analysis across three tumor types with >1 study and ≥2 trial arms. confirmed the strong correlation of 0.84 between PFS2 and OS (Table 3, Figure 3).

**Table 3:** Spearman's rank correlation based on pooled data from three tumors (breast, CRC, and NSCLC) with multiple study arms.

	**Correlation**	**95% CI**	***P*-value**
Fixed-effect model	0.9218	0.9145; 0.9292	<10^−4^
Random-effects model	0.8400	0.7160; 0.9641	0.0001

### Sensitivity and Publication Bias Analysis

By excluding any one study, we identified that the combined correlation between PFS2 and OS was still strong and did not vary substantially. To create a visual representation, a funnel plot was created showing the estimated correlation (plotted on the horizontal axis) vs. the reciprocal of its standard error (plotted on the vertical axis). As seen in Figure 3, there appears to be a slight asymmetry of the funnel plot and the published findings are close and scattered on either side of the funnel plot suggesting small systematic bias. However, despite this potential bias all the data show effects of strong correlation in the same direction. Moreover, Begg's, and Egger's tests, other standard formal tests for the presence of publication bias that avoid subjective interpretation as required for the funnel plot, showed no significant publication bias for the model (Begg's test, *P* = 0.3333 and Egger's test, *P* = 0.2626).

## Discussion

To our knowledge, this is the first publication to evaluate the correlation between PFS2 and OS in multiple solid tumor types. We found a strong correlation across all 15 studies and 28 study arms with Kendall's Tau correlation coefficient of 0.70 and a Pearson's correlation of 0.9 among randomized trials. A strong correlation was also observed, on meta-analysis of data from three indications represented by a total of ≥3 arms in multiple randomized trials (Spearman's rank correlation of 0.84). The consistently robust correlations observed in individual tumor types as well as pooled data from multiple indications suggest that in the absence of OS data, PFS2 can provide reassurance that treating with an experimental agent first followed by a second therapy is better than treating with standard of care therapy followed by second therapy. The observation underscores the importance of early treatment with active therapy that results in a sustained clinical benefit even after discontinuing the experimental therapy.

The clinical studies included in our analysis (Table 1) demonstrated median OS <5 yrs. However, treatment of early stage malignant diseases may improve survival beyond 10 years (31–34), and in such cases there is an unmet need to use endpoints like PFS2 that indicate/predict (potential) long term benefit. In the past many trials could not be implemented until the OS data matured as a result these therapies took years to benefit patients.

Moreover, in studies where results may be biased by factors that affect the next treatment in a sequence, PFS2 may be a more accurate measure of treatment benefit than PFS1. This possibility was tested in a recent analysis of data from PROREPAIR-B that included 419 patients with mCRPC with or without DNA repair germline mutations. PFS2 was significantly associated with OS, whether the PFS2 was clinical, radiographic or biochemical and was a better indicator of treatment benefit than PFS (35).

Analysis of correlations within indications as well as pooled data from multiple tumor types is strength of this analysis. Moreover, the population represented comprised 3,368 patients and seven indications. We found no evidence of publication bias. However, it is important to acknowledge that PFS2 can only be evaluated for patients who initiate a second treatment on study.

This analysis has several limitations that require caution in interpreting and generalizing the results. The results are based on trial level data rather than individual patient level data. The need to impute missing data is a limitation as is the variability introduced by differences in treatments and trial designs across studies.

Also, several questions regarding the correlation between PFS2 and OS are not addressed by this analysis and may warrant further investigation. Although we found a strong correlation across multiple tumor types and different treatments, factors such as the position of a specific study treatment in the sequence of therapies as well as any treatment-free interval between agents may affect the correlation. Lastly, we cannot rule out the possibility that the extent of the relationship between PFS2 and OS may vary among tumor types and therapies, especially in the rapidly evolving treatment landscape.

## Conclusions

A strong correlation was found between PFS2 and OS in multiple solid tumor clinical trials. These findings warrant further analysis to determine whether PFS2 is an appropriate surrogate endpoint for OS.

## Data Availability Statement

All datasets presented in this study are included in the article/supplementary material.

## Author Contributions

All authors participated in conception and design of this work, acquisition and collection of data, analysis of data, and participated in writing and reviewing this manuscript.

## Conflict of Interest

SC has a consulting/advisory relationship with Astellas Pharma, Bayer, Beigene, Clovis Oncology, Janssen Pharmaceuticals, and Pfizer; is a scientific advisory board member for Janssen Pharmaceuticals and Pfizer; has received honoraria from Clovis Oncology and Novartis; and has received research funding from Clovis Oncology and Sanofi/Aventis. AG is employed by and has ownership interests in IDEA Pharma; and has a consulting/advisory relationship with Pfizer and Janssen Pharmaceuticals. PM is employed by, has ownership interests in, and is an intellectual property rights/inventor/patent holder for XING Technologies P/L; and has received honoraria from Pfizer and Janssen Pharmaceuticals. EP is employed by and has ownership interests in IDEA Pharma; has ownership interests in Pfizer; and has a consulting/advisory relationship with Janssen Pharmaceuticals, Inc. The authors declare that this study received funding from Janssen Research and Development. SM, MW, and LZ are employed by (and have ownership interests in) Janssen R&D and were involved in all aspects of the review, statistical analysis, and manuscript preparation. This funding also supported a portion of the data search and editorial support.
